# A novel ferroptosis-related gene signature for predicting prognosis in multiple myeloma

**DOI:** 10.3389/fonc.2023.999688

**Published:** 2023-02-10

**Authors:** Dandan Gao, Rui Liu, Yang Lv, Yuandong Feng, Fei Hong, Xuezhu Xu, Jinsong Hu, Aili He, Yun Yang

**Affiliations:** ^1^ Department of Hematology, The Second Affiliated Hospital of Xi’an Jiaotong University, Xi’an, China; ^2^ Department of Cell Biology and Genetics, Xi’an Jiaotong University Health Science Center, Xi’an, China; ^3^ National-Local Joint Engineering Research Center of Biodiagnostics and Biotherapy, The Second Affiliated Hospital of Xi’an Jiaotong University, Xi’an, China

**Keywords:** multiple myeloma, ferroptosis, risk signature, tumor immunity, drug sensitivity

## Abstract

**Background:**

Multiple myeloma (MM) is a highly malignant hematological tumor with a poor overall survival (OS). Due to the high heterogeneity of MM, it is necessary to explore novel markers for the prognosis prediction for MM patients. Ferroptosis is a form of regulated cell death, playing a critical role in tumorigenesis and cancer progression. However, the predictive role of ferroptosis-related genes (FRGs) in MM prognosis remains unknown.

**Methods:**

This study collected 107 FRGs previously reported and utilized the least absolute shrinkage and selection operator (LASSO) cox regression model to construct a multi-genes risk signature model upon FRGs. The ESTIMATE algorithm and immune-related single-sample gene set enrichment analysis (ssGSEA) were carried out to evaluate immune infiltration level. Drug sensitivity was assessed based on the Genomics of Drug Sensitivity in Cancer database (GDSC). Then the synergy effect was determined with Cell counting kit-8 (CCK-8) assay and SynergyFinder software.

**Results:**

A 6-gene prognostic risk signature model was constructed, and MM patients were divided into high and low risk groups. Kaplan-Meier survival curves showed that patients in the high risk group had significantly reduced OS compared with patients in the low risk group. Besides, the risk score was an independent predictor for OS. Receiver operating characteristic (ROC) curve analysis confirmed the predictive capacity of the risk signature. Combination of risk score and ISS stage had better prediction performance. Enrichment analysis revealed immune response, MYC, mTOR, proteasome and oxidative phosphorylation were enriched in high risk MM patients. We found high risk MM patients had lower immune scores and immune infiltration levels. Moreover, further analysis found that MM patients in high risk group were sensitive to bortezomib and lenalidomide. At last, the results of the *in vitro* experiment showed that ferroptosis inducers (RSL3 and ML162) may synergistically enhance the cytotoxicity of bortezomib and lenalidomide against MM cell line RPMI-8226.

**Conclusion:**

This study provides novel insights into roles of ferroptosis in MM prognosis prediction, immune levels and drug sensitivity, which complements and improves current grading systems.

## Introduction

Multiple myeloma (MM), the second common hematologic malignancy, is characterized by clonal expansion of abnormal plasma cells (1, 2). The cardinal clinical manifestations of MM include bone lesions, anemia, hypercalcemia and renal failure. The incidence of MM is approximately 4.5-6 cases in every 100,000 people (3), which has been rising rapidly worldwide due to the aged tendency of population (4). Up to now, MM is still an incurable disease. MM patients have a median survival time of five years (5), and the study showed that there were about 11000 deaths annually in United States (6). Over the past ten years, there are multiple regimens, such as proteasome inhibitors (PIs), immunomodulatory drugs, CAR-T therapy and CD38 monoclonal antibody, which have improved the survival of MM patients significantly (7). However, most of MM patients finally go into relapse or drug resisitance (8). Thus, it is urgent to identify novel prognostic biomarkers and therapeutic targets of MM for better outcomes.

Ferroptosis, is a novel form of regulated cell death, differing from cell apoptosis, necrosis, autophagy, necroptosis, pyroptosis, which was first reported by Dr.Brent R.Stockwell in 2012 (9). Iron-dependent, excess reactive oxygen species (ROS) and lethal lipid peroxidation accumulation are typical features of ferroptosis (9). Ferroptotic cells will undergo cell membrane rupture, reduced mitochondrial volume, increased membrane density and absence of mitochondrial cristae when treated by ferroptosis inducer-erastin. Numerous genes have been shown to regulate cellular ferroptosis sensitivity, which could be divided into the ferroptosis driver group and ferroptosis suppressor group. Yang et al. showed that glutathione peroxidase 4 (GPX4) is a central regulator of ferroptosis and that ferroptosis can be induced by GPX4 knockout in mouse tumor xenografts (10). The transcription factor nuclear factor erythroid 2-related factor 2 (NRF2) is considered an important regulator of the antioxidant response and controlling the expression of various genes that involved redox homeostasis, such as xCT and GPX4, two of the most critical targets whose inhibition initiates ferroptosis (11). The p53 protein, which we all known serving as a critical tumor suppressor, could mediate cell cycle arrest, senescence and apoptosis, previous study found that p53 could inhibit cystine uptake and sensitize cells to ferroptosis by repressing expression of SLC7A11 (a component of the cystine/glutamate antiporter) (12). These genes also participate in various metabolic pathways, including iron metabolism, cysteine metabolism, lipid metabolism, as well as glucose metabolism (13, 14).

Studies revealed ferroptosis was associated with many diseases, such as neurological disorders, kidney injury, ischemia reperfusion injury and hematological diseases and cancers (15). Basuli et al. reported that compared with normal ovarian tissues, low ferroportin (FPN) expression and high transferrin receptor-1 (TFR1) and transferrin (TF) expression resulted in elevated iron levels and inhibited tumor proliferation (16). In hepatocellular carcinoma, CDGSH iron sulfur domain 1 (CISD1) was found to negatively regulate ferroptosis by inhibiting mitochondrial iron uptake, lipid peroxidation (17). Moreover, Sun et al. found that NRF2 and metallothionein 1G (MT1G) protected tumor cells from sorafenib-induced ferroptosis (18). In acute myeloid leukemia, upregulation of GPX4 negatively regulated ferroptosis and correlated with poor prognosis (19).

However, roles of ferroptosis in MM has not been fully elucidated. In this study, we constructed a multi-FRGs risk signature model for MM prognosis prediction and explored underlying mechanisms of ferroptosis in MM progression *via* bioinformatics methods, which provides novel insights for the prognostic biomarkers and therapeutic targets of MM.

## Materials and methods

### Data collection from publicly available databases

RNA-seq data and clinical information of all samples were obtained from the NCBI GEO databases (https://www.ncbi.nlm.nih.gov/geo/). The raw data was normalized and transformed by log2 using the “scale” method provided in the “limma” R package (version 4.0.3). Among them, the GSE47552 and GSE6477 were utilized for the identification of differentially expressed FRGs. The GSE9782 was applied as the training cohort for prognosis model construction, and the GSE24080 and GSE57317 were used as external validation cohorts, all of them had complete gene expression profiles as well as survival data. Then, a total of 107 FRGs were retrieved from Kyoto Encyclopedia of Genes and Genomes (KEGG, https://www.genome.jp/kegg/) and prior literatures (9, 20, 21).

### Identification of differentially expressed genes

The “limma” R package was used to identify the DEGs, with false discovery rate (FDR) < 0.05. The heatmap was performed by the “heatmap” R package (version 1.20.0).

### Functional enrichment analysis

Gene Ontology (GO) and KEGG analysis were conducted using the “clusterProfiler” R package (version 4.4.1) based on the DEGs (|log2FC| ≥ 1, FDR < 0.05) between the high and low risk groups. P values were adjusted with the BH method. Gene set enrichment analysis (GSEA) was carried out by using GSEA software (version 4.2.3). A p-value cutoff of 0.05 with a false discovery rate (FDR q-value) < 0.05 was considered statistically significant.

### PPI network construction and correlation analysis

String Database (http://string-db.org) (22) was used to construct protein-protein interaction (PPI) networks. The interaction threshold was set at 0.4. Correlation analysis was performed to demonstrate the association among different FRGs based on the Spearman’s correlation coefficient. The “corrgram” R package (version 1.14) was used for visualization.

### Construction and verification of the FRGs risk signature

We used univariate Cox regression analysis to identify prognostic FRGs among 107 FRGs with a threshold of p<0.05. To minimize overfitting risk, the LASSO-penalized Cox regression analysis was conducted to establish interested genes for use in the risk signature. Risk features for prognosis were determined using “glmnet” (version 4.1-4) and “survival” packages (version 3.3-1) (23). Optimal values of penalty parameter lambda were determined by 1k-fold cross-validation *via* the minimum criteria (24). Finally, MM patients’ risk scores were calculated based on normalized levels for each FRG and its regression coefficients using the formula: score = sum (corresponding coefficient × each gene’ s expression). The median value of the risk score was used to stratify patients into low and high risk groups. Two-sided log-rank tests and Kaplan-Meier survival analyses were performed to determine differences in OS between the two groups. ROC curve analysis assessed the model’s prognostic accuracy by “survivalROC” R packages (version 1.0.3). Based on the expression of genes in the risk signature, PCA was carried out with the “prcomp” function of the “stats” R package. Besides, t-SNE were performed to explore the distribution of different groups using the “Rtsne” R package.

### Immune infiltration

To assess immune infiltration, we evaluated the abundance of stromal cells and immune cells based on Estimation of Stromal and Immune cells in malignant tumors using Expression (ESTIMATE, https://sourceforge.net/projects/estimateproject/) data (25), a method that calculating immune score, stromal score and tumor purity of each sample for preliminary evaluation. The infiltrating scores of immune cells and the activity of immune-related pathways were assessed with single-sample gene set enrichment analysis (ssGSEA) (26) in the “gsva” R package (version 1.44.0). The annotated file of related immune pathways was provided in **Supplementary Table S1**.

### Prediction of drug sensitivity

We used the “pRRophetic” package (27) on R to predict drug sensitivity for each patient in the above cohort based on the Genomics of Drug Sensitivity in Cancer (GDSC) database (https://www.cancerrxgene.org/). The IC50 of the particular drug was estimated through ridge regression, while prediction accuracy was determined through 10-fold cross-validation using the GDSC training set. For all parameters, including “combat”, default values were obtained for removal of batch effect and tissue type of “BLOOD”, and duplicate gene expression was summarized as mean value (28).

### Cytotoxicity assay and synergy determination with SynergyFinder

The human multiple myeloma cell lines RPMI-8226 were seeded into 96-well plates at 1×10^4^ cells per well and were further treated as described below. Either single drugs or combinations were analyzed at the indicated amounts. After 24 h of treatment, the Cell counting kit-8 (CCK-8, 7 sea, Shanghai, China) was utilized to detect the cell viability and 10μl of CCK-8 solution was added to each well and the plates continued incubating for 1-4 h at 37°C. Finally, Thermo Scientific™ Multiskan™ FC was used to detect the optical density (OD) values at a wavelength of 450 nm. The half maximal (50%) inhibitory concentration (IC50) values were calculated by Prism 6.0 (GraphPad, La Jolla, CA, USA). The online SynergyFinder software (https://synergyfinder.fimm.fi) was used to calculate drug synergy scoring with the “inhibition index” (the inhibition index = 100 - Cell Viability) by the response surface model and the highest single agent (HSA) calculation method (29, 30). HSA Synergy scores greater than 0 were considered synergism (red regions) (31). Heatmaps of drug combination responses were also plotted to assess the therapeutic significance of the combination.

### Statistical analysis

The data have normal distribution (Shapiro-Wilk test for normality) and equal variances (Levene’s test for homogeneity of variances), then the t-test was used to compare and non-parametric for those were not, such as the Mann-Whitney test and the Wilcoxon test. All statistical analyses were performed with Prism 6.0 (GraphPad, La Jolla, USA). Pearson chi-square test was employed to compare the categorical variables. The Kaplan-Meier curve with a two-sided log-rank test was applied to compare the OS of patients between subgroups. Univariate and multivariate Cox regression analyses were conducted to figure out the independent factors related to survival rate. The Mann-Whitney test was used to compare the scores of infiltrating immune cells and the activities of immune-related pathways between low and high risk groups. All statistical analyses were executed utilizing R v4.1.2.

## Results

### Identification of differentially expressed FRGs

**Figure 1:**
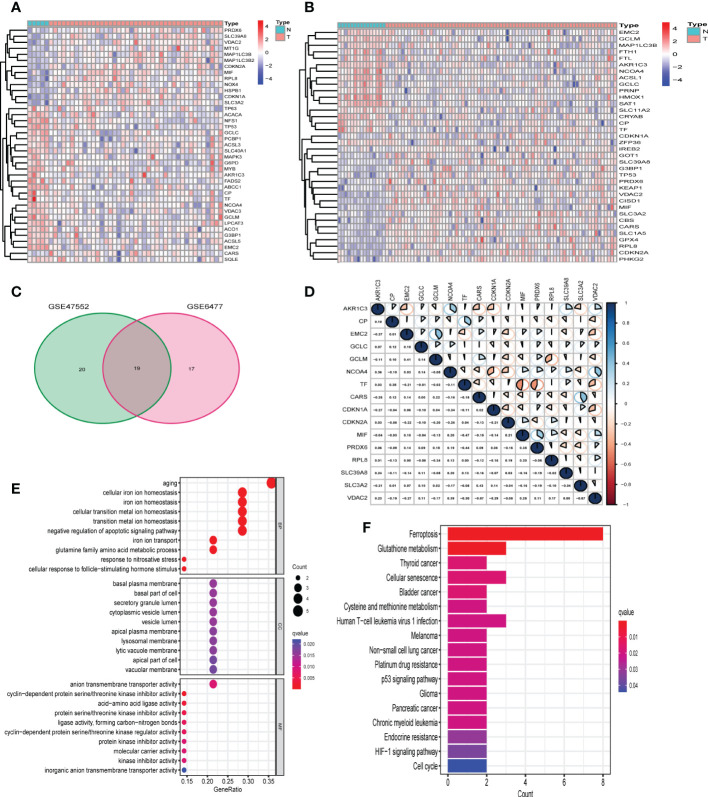
Identification of differentially expressed FRGs in GEO cohorts. **(A)** 39 FRGs were differentially expressed in GSE47552. **(B)** 36 FRGs were differentially expressed in GSE6477. **(C)** The venn plot of overlapped FRGs. **(D)** The correlation analysis of 16 differentially expressed FRGs in GSE6477 (blue: positive correlation; red: negative correlation; the larger the area of the sector, the correlation coefficient is closer to 1 or -1). **(E, F)** Bubble graph for GO enrichment and barplot graph for KEGG pathways.

The workflow chart of our study is shown in **Supplementary Figure 1**. The data of GSE47552 (n= 46, including 5 healthy donors and 41 NDMM patients) and GSE6477 (n= 88, including 15 healthy donors and 73 NDMM patients) were used for the identification of differentially expressed FRGs. 39 and 36 of 107 FRGs were identified as differentially expressed FRGs between healthy donors and NDMM in GSE47552 and GSE9782, respectively (**Figures 1A, B**). The volcano plots are shown in **Supplementary Figures 2A, B**. Then we used venn plot to get the 19 overlapped differentially expressed FRGs for further study (**Figure 1C**). Among them, 7 FRGs were down-regulated in NDMM (AKR1C3, CP, EMC2, GCLC, GCLM, NCOA4 and TF), and 9 FRGs were up-regulated (CARS, CDKN1A, CDKN2A, MIF, PRDX6, RPL8, SLC39A8, SLC3A2 and VDAC2), while the expression of 3 FRGs were differently expressed in the two cohorts (G3BP1, MAP1LC3B and TP53). To further ascertain the correlation of these differentially expressed FRGs, we conducted correlation analysis (**Figure 1D**). As figure showed that the expression of CARS was positively correlated with SLC3A2 (correlation coefficient = 0.41, p < 0.05), while the expression of TF was negatively correlated with MIF (correlation coefficient = -0.47, p < 0.05). The PPI network was in **Supplementary Figure 2C**, which illustrated the tight interactions of these genes. Furthermore, functional enrichment analysis was used to explore the biological functions and pathways of the above 16 intersection FRGs. The GO results indicated that these FRGs were enriched in iron-related terms, such as cellular iron ion homeostasis and cell redox homeostasis (**Figure 1E**). KEGG analysis also showed that ferroptosis and glutathione metabolism were closely enriched (**Figure 1F**).

### Identification of prognostic differentially expressed FRGs

**Figure 2:**
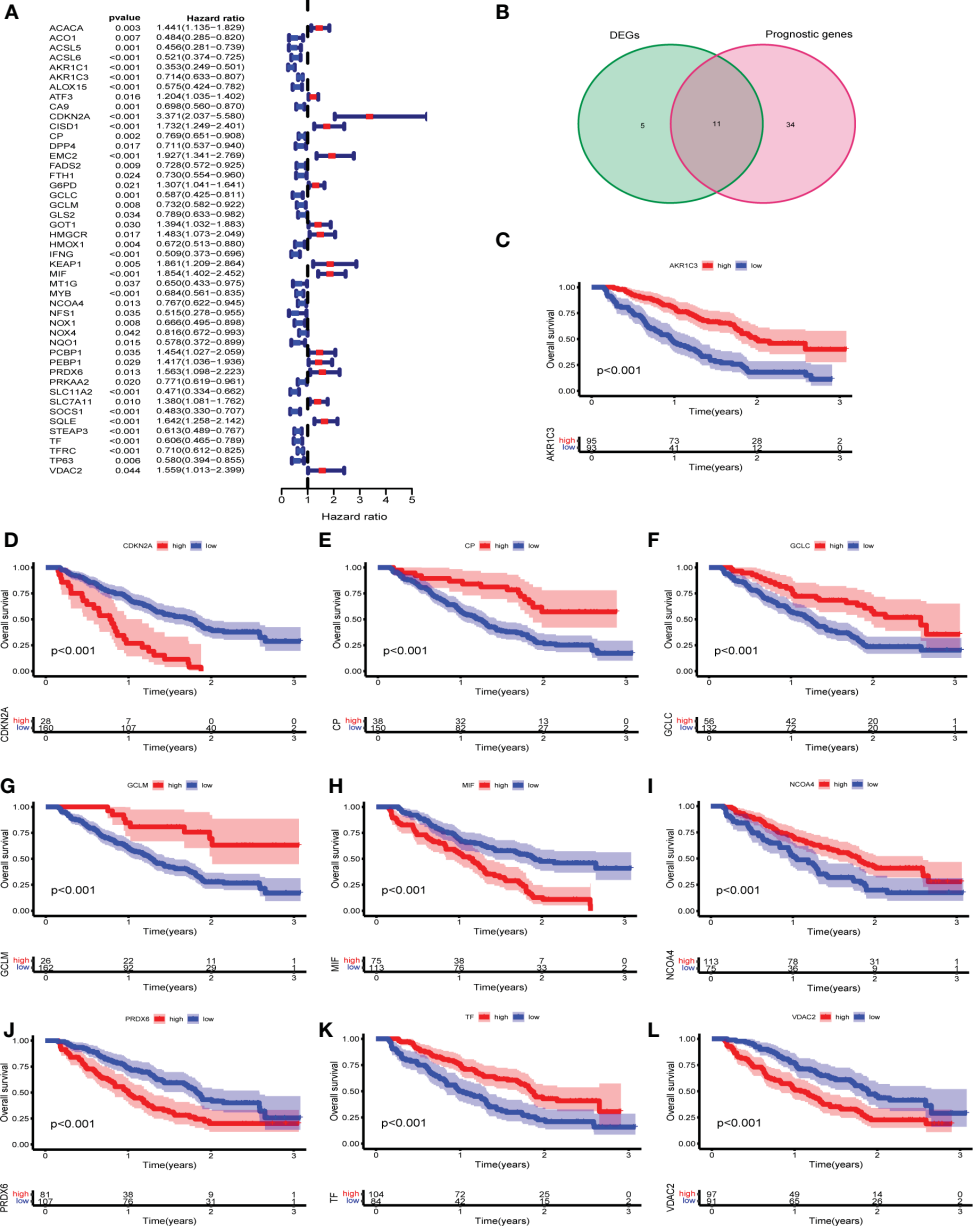
Identification of prognostic differentially expressed FRGs in GEO cohorts. **(A)** Univariate Cox regression in GSE9782. **(B)** The venn plot of DEGs and prognostic FRGs. **(C-L)** Kaplan-Meier survival curves of 10 intersection of genes for the OS of MM patients in GSE9782.

First, univariate Cox regression analysis was performed to analyze the 107 FRGs in GSE9782 (n= 188). The forest plot revealed that 45 FRGs were correlated with the OS of MM patients (p < 0.05) (**Figure 2A**). Then, we found the 11 intersection of genes with differentially expressed FRGs and prognostic FRGs (**Figure 2B**). Among them, CDKN2A, MIF, PRDX6 and VDAC2 were classified as risk-associated FRGs (HR>1), while AKR1C3, GCLC, GCLM, CP, NCOA4 and TF were classified as protective FRGs (HR<1). However, EMC2 was controversial, which was a risk-associated gene but was down-regulated in MM patients. Kaplan-Meier survival curves were plotted based on expression levels (high and low expression) of 10 FRGs. The OS of MM patients in the CDKN2A, MIF, PRDX6 and VDAC2 high expression groups was lower than that of patients in the low expression groups (p < 0.001). Conversely, the OS of MM patients in the AKR1C3, GCLC, GCLM, CP, NCOA4 and TF low expression groups was lower than that of patients in the high expression groups (p < 0.001) (**Figures 2C-L**). The results revealed that these 10 FRGs were significantly correlated with OS of MM patients.

### Construction and validation of FRGs risk signature in GEO cohorts

**Figure 3:**
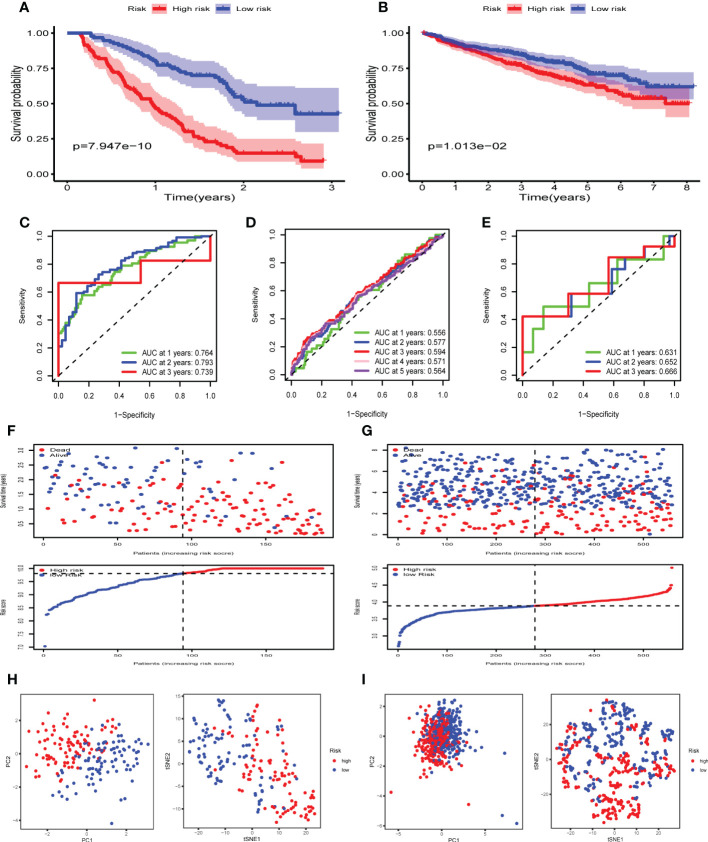
Construction and validation of FRGs risk signature in GEO cohorts. **(A, B)** Kaplan-Meier survival curves for the OS of MM patients in the high- and low-risk groups in GSE9782 **(A)** and GSE24080 **(B)**. **(C-E)** The time-dependent ROC curves for MM patients in GSE9782**(C)**, GSE24080 **(D)** and GSE57317**(E)**. **(F, G)** The risk plot and survival status for each MM patient in GSE9782 **(F)** and GSE24080 **(G)** with red dots being ceased cases and blue ones alive. **(H, I)** PCA and t-SNE analysis in GSE9782 **(H)** and GSE24080 **(I)**.

Next, LASSO regression analysis was performed to construct a prognostic model (**Supplementary Figure 3**). A total of 6 FRGs (AKR1C3, CDKN2A, CP, MIF, PRDX6 and TF) were identified and selected to develop a risk signature in GSE9782. The risk score for each MM patient could be computed using the following formula: the risk score = [(-0.24693 × expression of AKR1C3) + (0.96478 × expression of CDKN2A) + (−0.17386 × expression of CP) + (0.18016 × expression of MIF) + (0.40331 × expression of PRDX6) + (−0.08110 × expression of TF)]. According to the median risk score, 188 MM patients were divided into high and low risk groups. The Kaplan-Meier survival curve demonstrated that the OS of MM patients in the high risk group was significantly lower than that of the low risk group (p < 0.05) (**Figure 3A**). The time-dependent ROC curve showed the AUC was 0.764, 0.793 and 0.739 for survival rates of 1-, 2- and 3-year, respectively (**Figure 3C**). The risk plot presented an obvious separation of survival status between high and low risk MM patients. Patients with high risk scores exhibited significantly decreased survival rates (**Figure 3F**). PCA and t-SNE were performed to examine the risk score distribution differences between the low and high risk groups and the results showed that the patients of these two groups were distributed in two directions, suggesting our risk signature model could nicely distinguish the prognosis of MM patients (**Figure 3H**).

To validate the performance of the FRGs signature in predicting OS of MM patients, risk scores were calculated with the same formula for patients in two validation datasets, GSE24080 (n= 559) and GSE57317 (n= 55). Similarly, the Kaplan-Meier survival curve in GSE24080 also demonstrated that the high risk group showed a poor OS compared to the low risk group (p < 0.05) (**Figure 3B**). The time-dependent ROC curve in GSE24080 showed the AUC was 0.556, 0.577, 0.594, 0.571 and 0.564 for survival rates of 1-, 2-, 3-, 4- and 5-year, respectively (**Figure 3D**), while the time-dependent ROC curve in GSE57317 showed the AUC was 0.631, 0.652 and 0.666 for survival rates of 1-, 2- and 3-year, respectively (**Figure 3E**). The horizontal ordinate axis of the risk plot and survival event data were sorted according to the risk scores. Patients with high risk scores exhibited decreased survival rates and increased mortality rates in GSE24080 (**Figure 3G**). PCA and t-SNE were performed to demonstrate the significant risk score distribution differences between the low and high risk groups in the validation cohort (**Figure 3I**). Thus, these data implied that the FRGs risk signature had better capacity for predicting prognosis of MM patients.

### The ferroptosis risk score was the independent prognostic factor in MM

**Figure 4:**
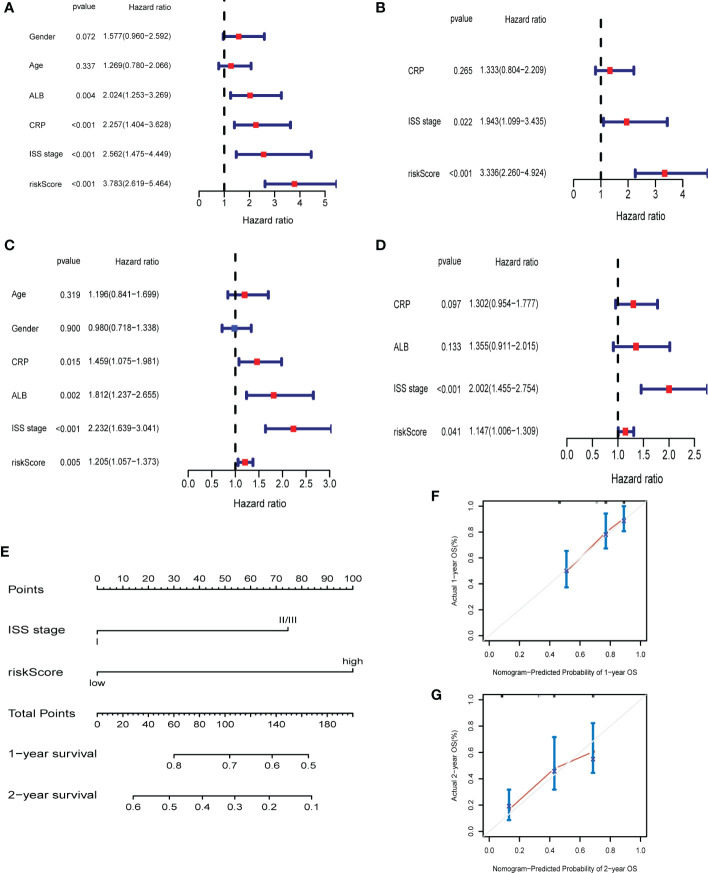
The ferroptosis risk score was the independent prognostic factor in MM. **(A, B)** Univariate and multivariate Cox regression in GSE9782. **(C, D)** Univariate and multivariate Cox regression in GSE24080. **(E)** Nomogram predicting 1- and 2-year OS of MM patients in the training cohort (GSE9782) based on the risk score and ISS stage. **(F, G)** Calibration plot of the Nomogram for 1- **(F)** and 2-year **(G)** OS in the training cohort (GSE9782).

Univariate Cox regression and multivariate Cox regression analysis were carried out to assess whether the risk score could serve as an independent and robust biomarker to predict OS of MM patients. In GSE9782 (training cohort), the univariate Cox regression analysis revealed that ALB, CRP, ISS stage and risk score were significantly correlated with OS (p < 0.05) (**Figure 4A**). Next, based on the multivariate analysis, ISS stage and risk score were confirmed as independent predictors for OS (p < 0.05) (**Figure 4B**). In GSE24080 (validation cohort), the similar results were obtained by univariate Cox regression analysis and multivariate analysis (**Figures 4C, D**). Then, the above two variables (ISS stage and risk score) in training cohort were used to construct the Nomogram for OS (**Figure 4E**). The calibration curves exhibited high consistency between the actual proportion of 1- and 2-year OS and the Nomogram-predicted probability (**Figures 4F, G**). Moreover, combination of risk score and ISS stage significantly improved prediction performance (**Supplementary Figure 4**). These results suggested that risk score could be a robust and reliable independent prognostic factor for OS of MM patients.

### Exploration of underlying mechanisms of the ferroptosis risk signature

**Figure 5:**
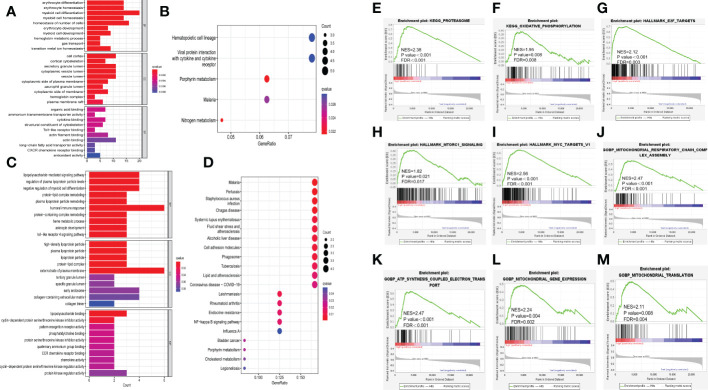
Exploration of underlying mechanisms of the ferroptosis risk signature. **(A, B)** Barplot graph of GO enrichment and bubble graph of KEGG pathways enrichment of DEGs in GSE9782. **(C, D)** Barplot graph of GO enrichment and bubble graph of KEGG pathways enrichment of DEGs in GSE24080. **(E-M)** GSEA results of KEGG pathways, HALLMARK and GOBP in GSE24080. NES, normalized enrichment score; FDR, false discovery rate.

We contrasted the gene expression pattern between high and low risk groups. 91 genes were identified significantly different between two risk groups in GSE9782. The heatmap, volcano plot and PPI network were shown in **Supplementary Figure 5**. Furthermore, we conducted GO and KEGG pathway enrichment analysis on these DEGs. For GO-Biological process (BP), DEGs were significantly enriched in myeloid cell development, differentiation and homeostasis, erythrocyte development, differentiation and homeostasis and ion homeostasis. The GO-Cellular Component (CC) analysis indicated that DEGs were mainly enriched in secretory granule lumen and vesicle lumen. For GO-Molecular Function (MF), DEGs mainly enriched in cytokine binding, toll-like receptor binding, actin binding and antioxidant activity (**Figure 5A**). The KEGG pathway analysis revealed that DEGs were mostly enriched in pathways in hematopoietic cell lineage and viral protein interaction with cytokine and cytokine receptor (**Figure 5B**). In GSE24080, DEGs were also enriched in humoral immune response (**Figure 5C**) and NF-kappa B signaling pathway (**Figure 5D**).

Moreover, GSEA was performed between high and low risk groups in GSE24080. The results revealed that high risk group was significantly active in proteasome (NES = 2.38, p < 0.001, FDR < 0.001, **Figure 5E**) and oxidative phosphorylation (NES= 1.95, p = 0.008, FDR = 0.008, **Figure 5F**) in KEGG gene set; E2F targets (NES = 2.12, p < 0.001, FDR = 0.003, **Figure 5G**), mTORC1 signaling (NES = 1.82, p = 0.021, FDR = 0.017, **Figure 5H**) and MYC targets (NES = 2.56, p < 0.001, FDR < 0.001, **Figure 5I**) in HALLMARK gene set; mitochondrial respiratory chain complex assembly (NES = 2.47, p < 0.001, FDR < 0.001, **Figure 5J**), ATP synthesis coupled electron transport (NES = 2.47, p < 0.001, FDR < 0.001, **Figure 5K**), mitochondrial gene expression (NES = 2.24, p = 0.004, FDR = 0.002, **Figure 5L**) and mitochondrial translation (NES = 2.11, p = 0.008, FDR = 0.004, **Figure 5M**) in GOBP gene set, others are showed in **Supplementary Figure 6**. These results implied that many immune-related pathways (such as cytokine binding and toll-like receptor binding) were significantly enriched in high risk group, thus we further aimed to evaluate the relationship between immune infiltration and ferroptosis risk signature.

### Immune infiltration was correlated with the ferroptosis risk signature

**Figure 6:**
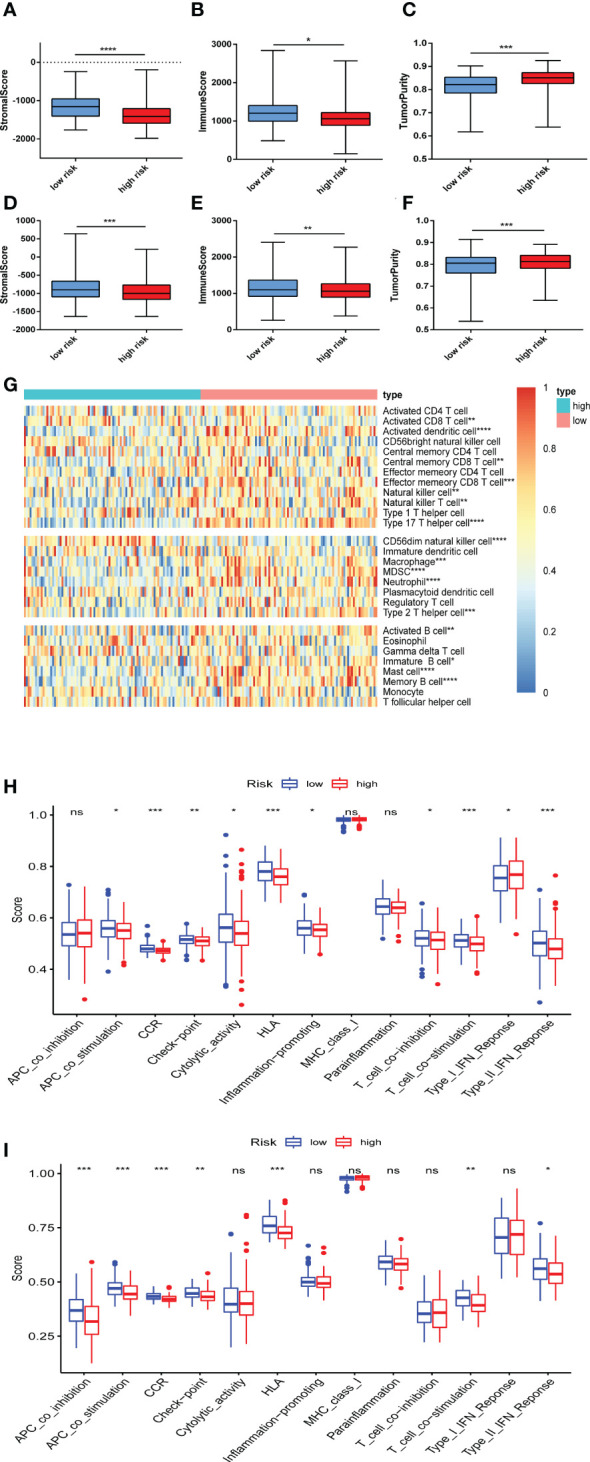
Immune infiltration was correlated with the ferroptosis risk signature. **(A-C)** The distribution of stromal score **(A)**, immune score **(B)**, and tumor purity **(C)** upon different risk score in GSE9782. **(D-F)** The distribution of stromal score **(D)**, immune score **(E)**, and tumor purity **(F)** upon different risk score in GSE24080. **(G)** The heatmap of the comparison in twenty-eight immune-related gene sets upon different risk score in GSE9782. **(H, I)** Thirteen immune-related functions and pathways were analyzed in patients with high and low risk score in GSE9782 **(H)** and GSE24080 **(I)**. ns, no significance; *p < 0.05, **p < 0.01, ***p < 0.001, ****p < 0.0001.

Based on the enrichment in immune response, we further used ESTIMATE algorithm to identify the differences in infiltrating immune cells between the high and low risk groups. The stromal scores (substrate cells in the tumor tissue) and immune scores (immune cell infiltration in the tumor tissue) were all significantly lower in the high risk group in GSE9782 (**Figures 6A, B**) and GSE24080 (**Figures 6D, E**). The scores of tumor purity were all higher in the high risk group in two cohorts (**Figures 6C, F**). Furthermore, ssGSEA was performed to evaluate the proportion of immune cells between the high and low risk groups in GSE9782. The results showed that activated CD8 T cell, activated dendritic cell, central memory CD8 T cell, effector memory CD8 T cell, natural killer cell, macrophage, neutrophil, type 2 T helper cell, activated B cell and memory B cell were all significantly decreased in the high risk group, which mostly participated in anti-tumor immune response. Conversely, the proportion of MDSC, an immunosuppressive cell, was reduced in the high risk group. Additionally, natural killer T cell, which played dual role in immune function, was also decreased in the MM patients with high risk score (**Figure 6G**). Moreover, corresponding immune functions and pathways were also different between the two risk groups (**Figures 6H, I**). The scores of APC co-stimulation, CCR, check point, HLA, T cell co-stimulation, type II IFN response were also lessened in the high risk group in GSE9782 (**Figure 6H**) and GSE24080 (**Figure 6I**). To sum up, MM patients with high risk scores showed significantly decreased immune infiltration levels and immune function.

### Drug sensitivity was associated with the ferroptosis risk signature

**Figure 7:**
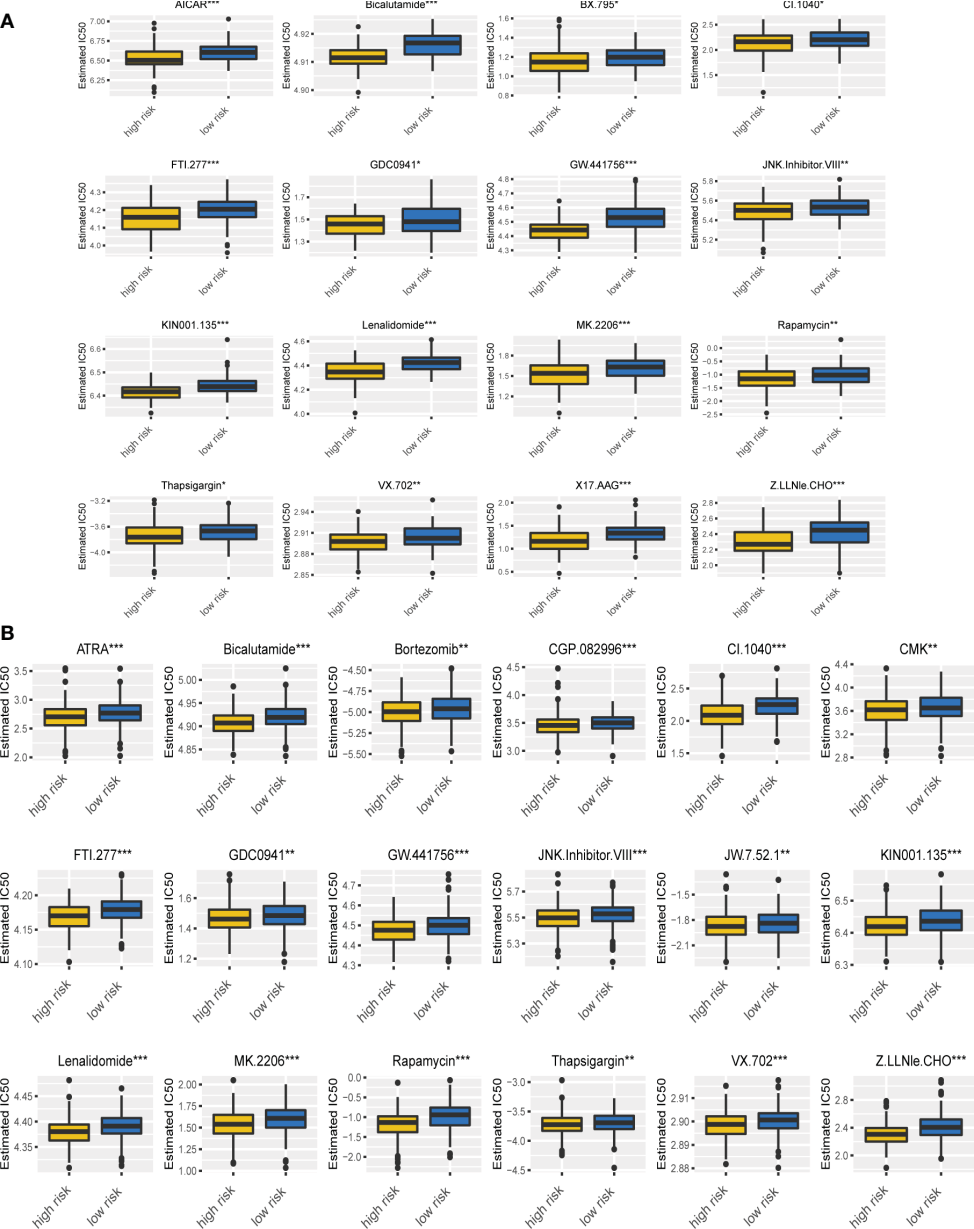
Drug sensitivity was associated with the ferroptosis risk signature. **(A, B)** The correlation of risk scores with the IC50 of various drugs on MM patients in GSE9782 **(A)** and GSE24080 **(B)**. *p < 0.05, **p < 0.01, ***p < 0.001.

To further explore the difference of drug sensitivity in the two risk groups, we compared the estimated IC50 levels of 129 drugs. Among those, 21 drugs showed decreased IC50 and higher sensitivity in high risk group (**Figures 7A, B**). Especially, the sensitivity of bortezomib and lenalidomide, which are important drugs and first-line therapy for MM treatment, were significantly increased in MM patients with high risk scores. Given that these two drugs have been used widely in clinical, we compared post-treatment status of MM patients with different risk scores based on GSE9782, in which patients were pretreated with bortezomib. As **Supplementary Figure 7** showed, MM patients in high risk group progressed significantly faster than low risk group (p = 0.031). Although MM patients with high risk scores were more sensitivity to bortezomib, it is essential to combine other therapies (such as lenalidomide, CD38 antibody, CAR-T cell etc.) to prolong progression-free survival. In addition, we found that the estimated IC50 of AICAR (Acadesine, AMPK activator), ATRA (all-trans-retinoicacid), GDC0941 (PI3K inhibitor), JNK.Inhibitor.VIII, rapamycin (mTOR inhibitor) and thapsigargin (ATPase inhibitor) were also lower in MM patients in the high risk group based on our risk signature. Altogether, these findings implied that the ferroptosis signature is tightly correlated with drug sensitivity. Therefore, the risk score might be a potential indicator for choosing appropriate drugs for MM individualized treatment.

### Validations for FRGs expressions and determination of synergy effect *in vitro*

**Figure 8:**
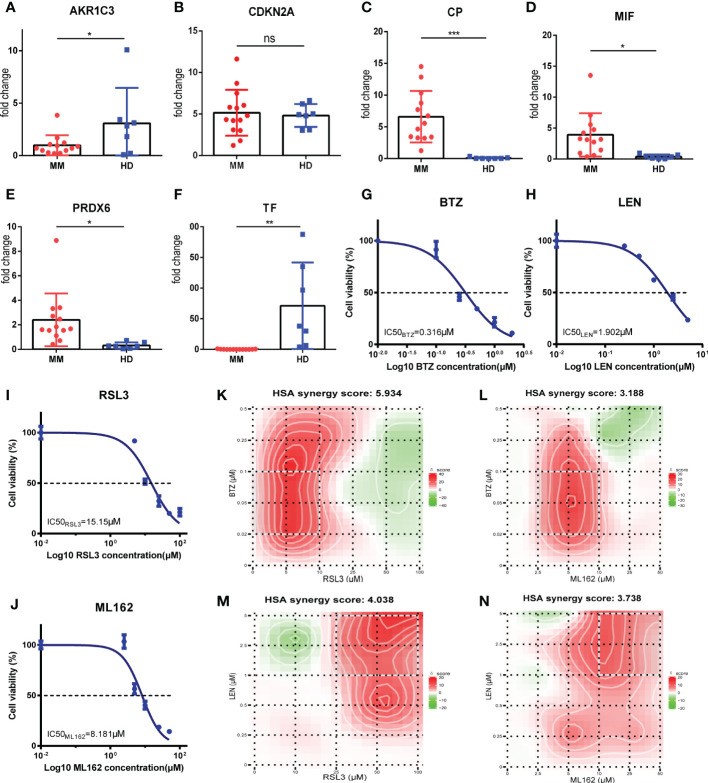
Validations for FRGs expressions and determination of synergy effect *in vitro*. **(A-F)** AKR1C3, CDKN2A, CP, MIF, PRDX6 and TF mRNA expression in MM patients (MM, n=13) *vs* healthy donors (HD, n=7). **(G-J)** Drugs at the indicated concentrations were used to treat cells for 24h, and cell viability was assessed by CCK-8 assay.Dose-response studies and the IC50 of BTZ **(G)**, LEN **(H)**, RSL3 **(I)**, ML162 **(J)** in RPMI 8226 cell line. Data are expressed as the means ± SD. **(K-N)** Heatmaps of drug combination responses. HSA synergy scores were calculated using Synergyfinder software. Scores > 0 indicated synergism. The HSA synergy scores of BTZ+RSL3 **(K)**, BTZ+ML162 **(L)**, LEN+RSL3 **(M)**, and LEN+ML162 **(N)** in RPMI 8226 cell line were 5.934, 3.188, 4.038, and 3.738, respectively. The gradation of the red regions indicates the intensity of synergism. The white rectangle indicates the concentrations encompassing the region of highest synergy, and the X- and Y-axes corresponding to the sides of the white rectangle indicate the concentrations at which the drug combination had the maximum effect on cell growth inhibition. MM, multiple myeloma; HD, healthy donors; BTZ, bortezomib; LEN, lenalidomide; HSA, highest single agent. *p<0.05, **p<0.01, ***p<0.001.

To further validate the results of bioinformatic analysis, we collected the bone marrow samples from 7 healthy donors (HD) and 13 MM patients and performed qRT-PCR to measure the mRNA levels of the relevant FRGs (AKR1C3, CP, CDKN2A, MIF, PRDX6 and TF), mostly consistent with the results which we have described before. The results demonstrated that the expression of CP, MIF and PRDX6 were elevated in MM patients. In addition, the expression of AKR1C3 and TF were decreased in MM patients (p<0.05). However, the expression level of CDKN2A in MM patients was higher than it in healthy donors, but no significant difference was observed (p>0.05) (**Figures 8A-F**). Similarly, MIF and PRDX6 were significantly upregulated in MM cell lines compared to normal bone marrow stromal cell lines HS5.And the expression levels of AKR1C3, CDKN2A, CP and TF in MM cell lines were statistically lower than these in HS5 cell line (**Supplementary Figures 8A-F**). The reason for the inconsistent results between clinical and cell lines may be the insufficient sample size. Increasing sample size may aid in obtaining positive results in future studies.

Subsequently, we performed a drug combination analysis to determine whether ferroptosis was associated with the sensitivity of bortezomib (BTZ) and lenalidomide (LEN) against MM cell line RPMI-8226. First, the CCK-8 assay was performed to measure the biological effect of BTZ, LEN, and two kinds of ferroptosis inducers (RSL3 and ML162) in the RPMI-8226 and dose-response curves were made. These four drugs showed concentration-dependent anti-proliferative effects, and the IC50 values were calculated and shown in **Figures 8G-J** (BTZ: 0.316μM; LEN: 1.902μM; RSL3: 15.15μM; ML162: 8.181μM). According to the new concentration gradient based on IC50 values and the corresponding inhibition index, the drug HSA synergy scores were calculated with the online SynergyFinder software. Indeed, treatment with ferroptosis inducers (RSL3 and ML162) and BTZ or LEN showed highly synergistic effects in inhibiting tumor proliferation (HSA synergy scores>0) (**Figures 8K-N**). As shown in the figures, the white rectangle indicates the region of the maximum synergistic area. And the dose-response matrix and 3D plot were in **Supplementary Figures 9A-H**. Collectively, these results of the *in vitro* experiment showed that ferroptosis inducers (RSL3 and ML162) may synergistically enhance the cytotoxicity of bortezomib and lenalidomide against MM, which may improve the prognosis of high risk MM patients.

## Discussion

Ferroptosis, an emerging type of regulated cell death, is widely studied in various diseases. Companied with iron overload, excess ROS and unrestricted lipid peroxidation, ferroptosis has been believed to play an essential role in tumorigenesis (9). In this article, we demonstrated the significance of ferroptosis regulators in MM and assessed their relationship with MM prognosis. First, we identified 10 differentially prognostic FRGs by taking the intersection of DEGs and prognostic FRGs. Then, lasso regression was carried out based on 10 intersected genes and a new 6 FRGs risk signature was established. By using the established formula and calculating risk score of every MM patient, we confirmed the appropriate cutoff value and divided patients into two risk groups (high and low risk groups) in training cohort (GSE9782) and two validation cohorts (GSE24080 and GSE57317). Kaplan-Meier survival curves showed that the OS of MM patients in high risk group was significantly shorter than in low risk group. Meanwhile, ROC curves verified the predictive efficiency of this risk signature. To determine the survival status of MM patients based on this model, the risk plot was performed by ranking risk score of every patient, and the results suggested that an obvious separation of survival status between two risk groups. Patients with high risk scores exhibited markedly decreased survival and increased death rates. Moreover, univariate and multivariate Cox regression analysis also manifested that the risk score was an independent prognostic factor.

In our model, six FRGs were included: AKR1C3, CDKN2A, CP, MIF, PRDX6 and TF. Aldo-keto reductase 1C3 (AKR1C3) is a member of superfamily of NAD(P)H-linked oxidoreductases, serving as a reductant to reduce aldehydes and ketones to alcohols (32). Recent studies have demonstrated that AKR1C3 was overexpressed in various cancers such as breast cancer (33), prostate cancer (34, 35) and acute myeloid leukemia (AML) (36–38). In AML, AKR1C3 may regulate myeloid and erythroid differentiation *via* prostaglandin D2 metabolism (39). Previous studies revealed that cyclin dependent kinase inhibitor 2A (CDKN2A) played an important role in oncogenesis and tumor progression. The loss or methylation of CDKN2A is relatively common in pretreatment follicular lymphoma biopsy specimens and correlated with poor outcome (40). Ceruloplasmin (CP), a kind of ferroxidases, could discharge iron from cells and regulate cellular iron homeostasis (41), but the roles of CP in tumors are controversial. The serum CP levels were elevated in lung cancer, colon carcinoma, epithelial ovarian cancer (42–44), and correlated with invasiveness of cancer cells, while in adrenocortical and hepatocellular carcinoma, the expression levels of CP were down-regulated (45, 46). Macrophage migration inhibitory factor (MIF) is a soluble pro-inflammatory cytokine (47). Wang et al. demonstrated that MIF expression was significantly higher in relapsed MM patients, and MM patients with higher MIF expression had poorer OS (48). Furthermore, knockout of MIF in MM cell lines sensitized the PIs-induced cell apoptosis *via* regulating SOD1 misfolding and loss of SOD1 activity (48). Peroxiredoxin 6 (PRDX6), the only 1-Cys member of PRDX family, is a multifunctional enzyme, including iPLA2 activity, LPCAT activity and glutathione peroxidase activity (49). PRDX6 has been reported to positively regulate oncogenesis and progression by activating the JAK2/STAT3 signaling pathway (50). Transferrin (TF), an iron-carrier protein, is essential for transporting iron and is required by all living organisms, especially highly proliferative cells, because of its requirement for DNA replication (51). *In vivo* experiment showed that TF was a growth factor in some tumors, such as leukemia, breast cancer and pituitary tumor (52–54). These six FRGs were reported expressed differently in various cancer types and the internal mechanism was overwhelmingly intricate. Therefore, the further study is urgently needed to be conducted in MM.

Next, based on the DEGs between the two risk groups, GO and KEGG enrichment analysis were performed. Interestingly, we found many immune-related biological processes and pathways were enriched in training and validation cohorts, such as cytokine binding, Toll-like receptor binding, CXCR chemokine receptor binding, humoral immune response, CCR chemokine receptor binding, chemokine activity and NF-kappa B signaling pathway. Then, immune infiltration level was measured *via* ESTIMATE algorithm and ssGSEA. The results indicated that MM patients with higher risk scores exhibited lower immune scores. Immune cell subsets were significantly different in high and low risk groups. Among them, the anti-tumor immune cells, such as activated CD8 T cell, central memory CD4 T cell, effector memory CD8 T cell, natural killer cell, natural killer T cell, type 17 T helper cell and type 2 T helper cell were reduced in high risk group, while the protumor immune cell, such as MDSC was also reduced in high risk group. Then, we compared immune-related pathways between the two risk groups. The results showed that T cell co-inhibition was positively correlated while cytolytic activity was negatively correlated with higher risk score, suggesting tumor immunity in high risk group was suppressed. These results reminded us that the potential connection between ferroptosis and tumor immunity. For instance, CDKN2A expression was correlated with infiltrating lymphocyte (TIL) levels in cancers, mainly involving in natural killer cell-mediated cytotoxicity pathways, antigen processing and presentation, olfactory transduction pathways, and regulation of the autophagy pathway in multiple cancers (55). Monocyte-derived MIF is reported centrally involved in human monocytic MDSC induction/immune suppressive function and that targeting MIF may provide a novel means of inducing anti-tumor responses in late stage melanoma patients (56). However, the APC co-inhibition and co-stimulation were different between two risk groups. The reason for this phenomenon maybe due to ferroptotic cells product many different signals and transmit them to antigen presentation cells, thus resulting in different effects, which including inhibition and activation (57). In general, we discovered that higher risk scores tightly correlate with the immunosuppression, which maybe the cause for poor prognosis of MM patients in high risk group.

Moreover, we conducted GSEA analysis based on this risk signature, and found that the pathway in proteasome, E2F targets and MYC targets were more active in high risk group, which correlated with tumorigenesis and drug resistance. Intriguingly, the mitochondrial activity was also elevated in MM patients with higher risk scores, including oxidative phosphorylation and mitochondrial genes expression, suggesting that higher energy demands were needed in tumor cells of MM patients in high risk group.

In the end, we assessed drug sensitivity based on this FRGs risk signature. MM patients with higher risk scores were predicted to be more sensitive to lenalidomide and bortezomib, which still the first line therapy for MM patients. Moreover, higher risk scores MM patients also presented a better sensitivity to ATAR, JNK inhibitor, rapamycin and thapsigargin. Interestingly, CCI-779 (also called temsirolimus), a derivative of rapamycin, is also an mTOR inhibitor and has been studied combined with bortezomib in relapsed or relapsed refractory multiple myeloma (NCT00483262). The results showed that the proportion of patients with a partial response or better was 33% (14 of 43; 90% CI 21-47) in the phase 2 study, suggesting mTOR inhibitors have the potential role in combination with bortezomib for the treatment of relapsed and refractory MM patients (58). Although there has been no study of other drugs for MM patients, the results could provide us novel insights into exploring new treatments in MM. Furthermore, we assessed the efficacy of combination ferroptosis inducers (RSL3 and ML162) with bortezomib or lenalidomide in MM cell line through an effect-based methodology (Synergyfinder). The results of the *in vitro* experiment showed that ferroptosis inducers may synergistically enhance the cytotoxicity of bortezomib and lenalidomide against MM, which may improve the prognosis of high risk MM patients.

Finally, there are some limitations in our study. First, our risk signature model was conducted and verified based on the public databases, further studies are needed to validate in clinical. Second, due to incomplete clinical information in GEO databases, our model could not assess the R-ISS stage or mSMART risk stratification in MM patients based on the risk signature. Third, the relationship between the risk signature and immune activity has not yet been experimentally addressed, which should be ascertained in the future.

## Conclusion

We defined a novel prognostic model of 6 FRGs in MM, including AKR1C3, CDKN2A, CP, MIF, PRDX6 and TF. The model could divide MM patients into high and low risk groups and accurately and stably predict the OS of MM patients. The underlying mechanism maybe correlated with impaired anti-tumor immunity in high risk group. Moreover, the ferroptosis risk score has potential application for MM individualized therapy. We believe that the 6 FRGs are potential prognostic biomarkers and therapeutic targets for MM.

## Data availability statement

The original contributions presented in the study are included in the article/**Supplementary Material**. Further inquiries can be directed to the corresponding authors.

## Author contributions

DG: Conceptualization, Investigation, Methodology, Software,Validation, Data curation, Formal analysis, Writing–original draft,Visualization. RL: Conceptualization, Investigation, Methodology,Software, Validation, Data curation, Formal analysis, Visualization. YL: Methodology, Validation, Data curation, Formal analysis, Investigation, Visualization. YF: Methodology, Validation, Data curation, Formal analysis, Investigation, Visualization. FH: Formal analysis. XX: Formal analysis. JH: Methodology, Investigation, Visualization. AH: Supervision, conceptualization, Validation. YY: Supervision, conceptualization, Validation.
