# Supplementary material for: A novel ferroptosis-related gene signature for predicting prognosis in multiple myeloma
Source: Front Oncol. 2023 Feb 10;13:999688. doi: 10.3389/fonc.2023.999688 (PMC9950937; doi:10.3389/fonc.2023.999688)
Supplement: Supplementary file 2 [file Table_2.docx]

**Table S2. Primer sequences used for Realtime PCR analysis.**

| **Gene symbol** | **Gen Bank Accession no.** | **Primer set sequence (5’->3’)** | **Amplicon size (bp)** |
| --- | --- | --- | --- |
| *AKR1C3(h)* | NM_001253908.2 | forward:  5'-AAGCTTTGGTCCACTTTTCATC-3'  reverse:  5'-GGTCAACATAGTCCAATTGAGC-3' | 88 |
| *CDKN2A(h)* | NM_058197.5 | forward:  5'-GATCCAGGTGGGTAGAAGGTC-3'  reverse:  5'-CCCCTGCAAACTTCGTCCT-3' | 74 |
| *CP(h)* | NM_000096.4 | forward:  5'-GGGCCAATGAAAATATGCAAGA-3'  reverse:  5'-CTGATCAGGTGCAGTTGTAAAC-3' | 156 |
| *MIF(h)* | NM_002415.2 | forward:  5'-CCGAGAAGTCAGGCACGTAG-3'  reverse:  5'-ATAGTTGATGTAGACCCTGTCCG-3' | 381 |
| *PRDX6(h)* | NM_004905.3 | forward:  5'-GACAGCTCGTGTGGTGTTTG-3'  reverse:  5'-TCAAAGTTCCTGCCAGTGGT-3' | 87 |
| *TF(h)* | NM_001063.4 | forward:  5'-GTGCAGTGTCGGAGCATGAG-3'  reverse:  5'-CGCTTCGTTTGCCGCAATG-3' | 146 |
| *β-actin(h)* | NM_001101.5 | forward:  5'-GAGCACAGAGCCTCGCCTTT-3'  reverse:  5'-TCATCATCCATGGTGAGCTGG-3' | 70 |
