# Supplementary material for: A novel ferroptosis-related gene signature for predicting prognosis in multiple myeloma
Source: Front Oncol. 2023 Feb 10;13:999688. doi: 10.3389/fonc.2023.999688 (PMC9950937; doi:10.3389/fonc.2023.999688)
Supplement: Supplementary file 3 [file Table_3.docx]

**Supplementary Table S3. Analysis of T lymphocyte subsets in clinical MM patients.**

| **Patient ID** | **CD3+CD4+(%)** | **CD3+CD8+(%)** | **CD4+/CD8+** |
| --- | --- | --- | --- |
| Patient#1 | 29.02 | 63.78 | 0.46↓ |
| Patient#2 | 35.56 | 24.68 | 1.44↓ |
| Patient#3 | 29.07 | 34.17 | 0.85↓ |
| Patient#4 | 37.66 | 44.37 | 0.85↓ |
| Patient#5 | 32.58 | 25.31 | 1.29↓ |
| Patient#6 | 48.79 | 33.84 | 1.44↓ |
| Patient#7 | 27.26 | 32.78 | 0.83↓ |
| Patient#8 | 30.31 | 49.76 | 0.61↓ |
| Patient#9 | 39.94 | 21.66 | 1.84- |
| Patient#10 | 28.02 | 54.27 | 0.52↓ |
| Patient#11 | 43.39 | 34.06 | 1.27↓ |
| Patient#12 | 27.02 | 38.7 | 0.7↓ |
| Patient#13 | 38.04 | 28.1 | 1.35↓ |

Reference value: CD3+CD4+ cells: 35-55%; CD3+CD8+ cells: 20-30%; CD4+/CD8+: 1.5~2.0.
