# Supplementary material for: A novel ferroptosis-related gene signature for predicting prognosis in multiple myeloma
Source: Front Oncol. 2023 Feb 10;13:999688. doi: 10.3389/fonc.2023.999688 (PMC9950937; doi:10.3389/fonc.2023.999688)
Supplement: Supplementary file 4 [file DataSheet_1.docx]

Supplementary Material

# Materials and Methods

## Subjects and chemicals

This work was approved by the institutional review board. All patients and healthy donors provided written informed consent of using their samples for research purposes. Bone marrow specimens were obtained from MM patients and healthy donors (HD) at the department of hematology, The Second Affiliated Hospital of Xi’an Jiaotong University. PBMCs were isolated from freshly blood with Ficoll density gradient centrifugation and then total cellular RNA were harvested, stored at -80°C. Bortezomib (HY-10227), Lenalidomide (HY-A0003), RSL3 (HY-100218A) and ML162 (HY-100002) were all purchased from MedChemExpress (Shanghai, China), and diluted to 10mM, 100mM, 5mM and 5mM as the final concentration, respectively.

## Cell lines and cell culture

The human multiple myeloma cell lines RPMI-8226, MM.1S, U266, NCI-H929, OPM2, Karpas 707 and human bone marrow stromal cell lines HS5 were obtained from Professor Jinsong Hu of Xi’an Jiaotong University Health Science Center (Xian, Shaanxi, China). MM cell lines were maintained in RPMI1640 (BasalMedium, China) with 10% fetal bovine serum (FBS, Biological Industries, Kibbutz Beit Haemek, Israel), 100 μg/ml L-glutamine and antibiotics. HS5 was maintained in high-sugar DMEM (BasalMedium, China) with 10% FBS, penicillin and streptomycin. All cells were incubated at 37°C in a humidified atmosphere with 5% CO_2_.

## Quantitative real-time polymerase chain reaction (qRT-PCR)

Total RNA was extracted from MM cells using TRIzol reagent (CWBIO, China). RNA concentrations were measured by Nanodrop and reverse-transcribed to cDNA using the cDNA Reverse Transcription Kit (CWBIO, China). qRT-PCR was performed using the SYBR Green PCR kit (CWBIO, China) and the three-step method was applied. Pre-denatured at 95°C for 5 min, denatured at 95°C for 10 s, annealed at 55–60°C for 20 s, and extended at 72°C for 20 s, the last three steps require 40 cycles. The primers are listed in Supplementary Table S2. β-Actin was used as control. The 2−ΔCt method was used for the qRT-PCR analysis.

# Results

**2.1 Validation of FRGs risk signature in different treatment subgroups in GSE24080**

In the dataset GSE24080, there were two subgroups with different treatment regimens, TT2 and TT3, where the chemotherapy agent in TT2 was thalidomide (n=351) and in TT3 was bortezomib (n=214). Within the subgroups, we also calculated the risk score for each patient according to the constructed risk signature and analyzed the relationship between risk score and overall survival of patients. We found that in the TT2 subgroup, the prognosis of the patients whose have higher risk scores was worse (Supplementary Figure 10A); however, in the TT3 subgroup, there was no significant relationship between this risk score and overall survival (Supplementary Figure 10B), which may be due to the smaller number of patients in the TT3 group (this is indeed a limitation of our study). Further, in the training group of this study, patients treated with bortezomib in GSE9782 (n=188), it can be seen that this risk score correlates with the prognosis of patients after treatment (Figure 3A). Taken together, we believe that this risk score to some extends can guide treatment and predict the patient’s outcome after treatment.

**2.2 Analysis of T lymphocyte subsets in clinical MM patients**

We analyzed the T lymphocyte subsets of 13 MM patients, whose bone marrow specimens were obtained to validate the expression of the FRGs. We found that the ratio of CD4+/CD8+ was significantly lower than 1.5 in 12 patients (92.3%) (CD4+/CD8+ reference value: 1.5-2.0), suggesting that the patients were in a state of immunosuppression (Supplementary Table S3). This further suggests higher risk scores tightly correlate with immunosuppression, which may cause the poor prognosis of MM patients in the high risk group.

# Supplementary Figures

#
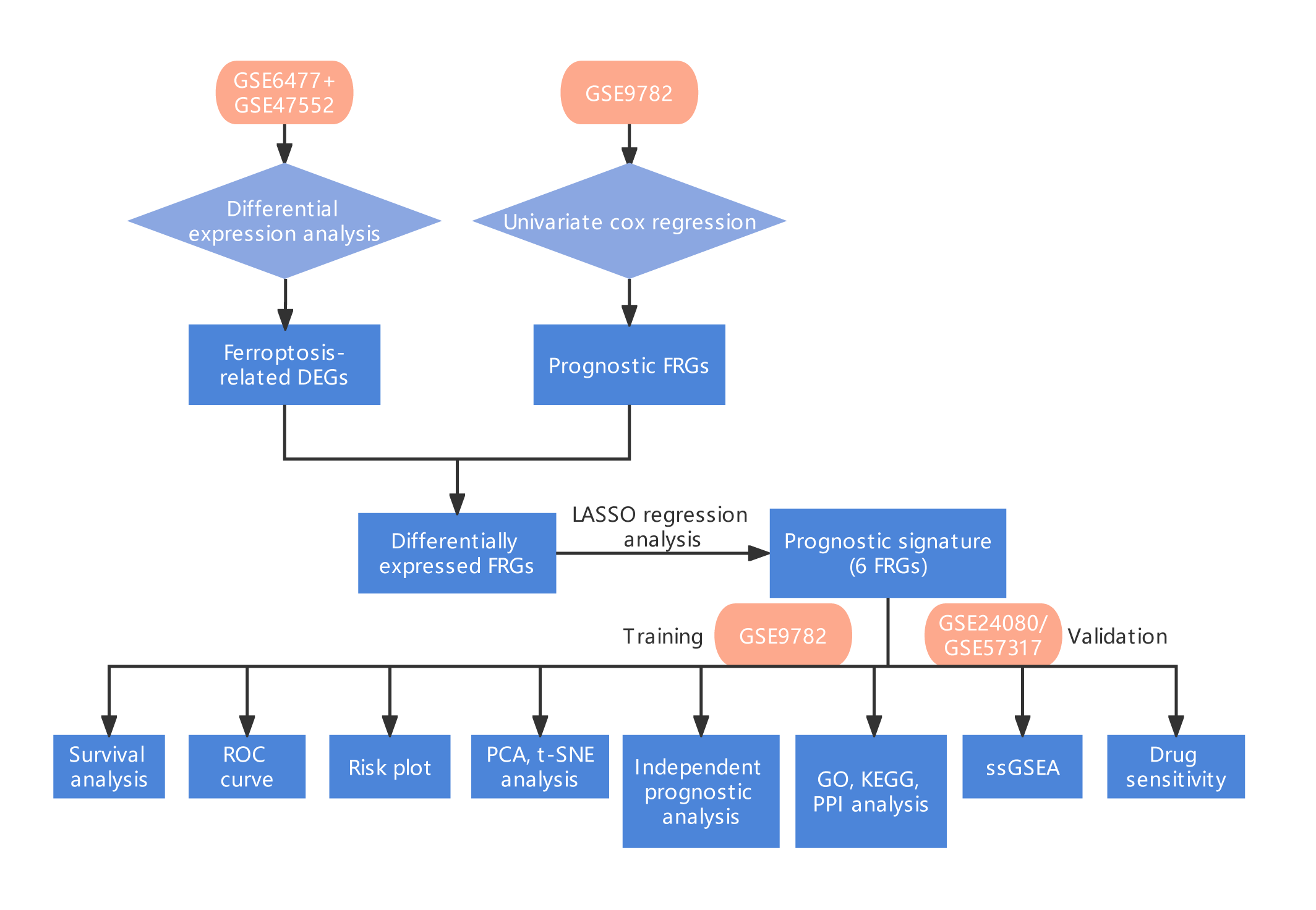
Supplementary Figure 1. The workflow chart of data collection and analysis in study.


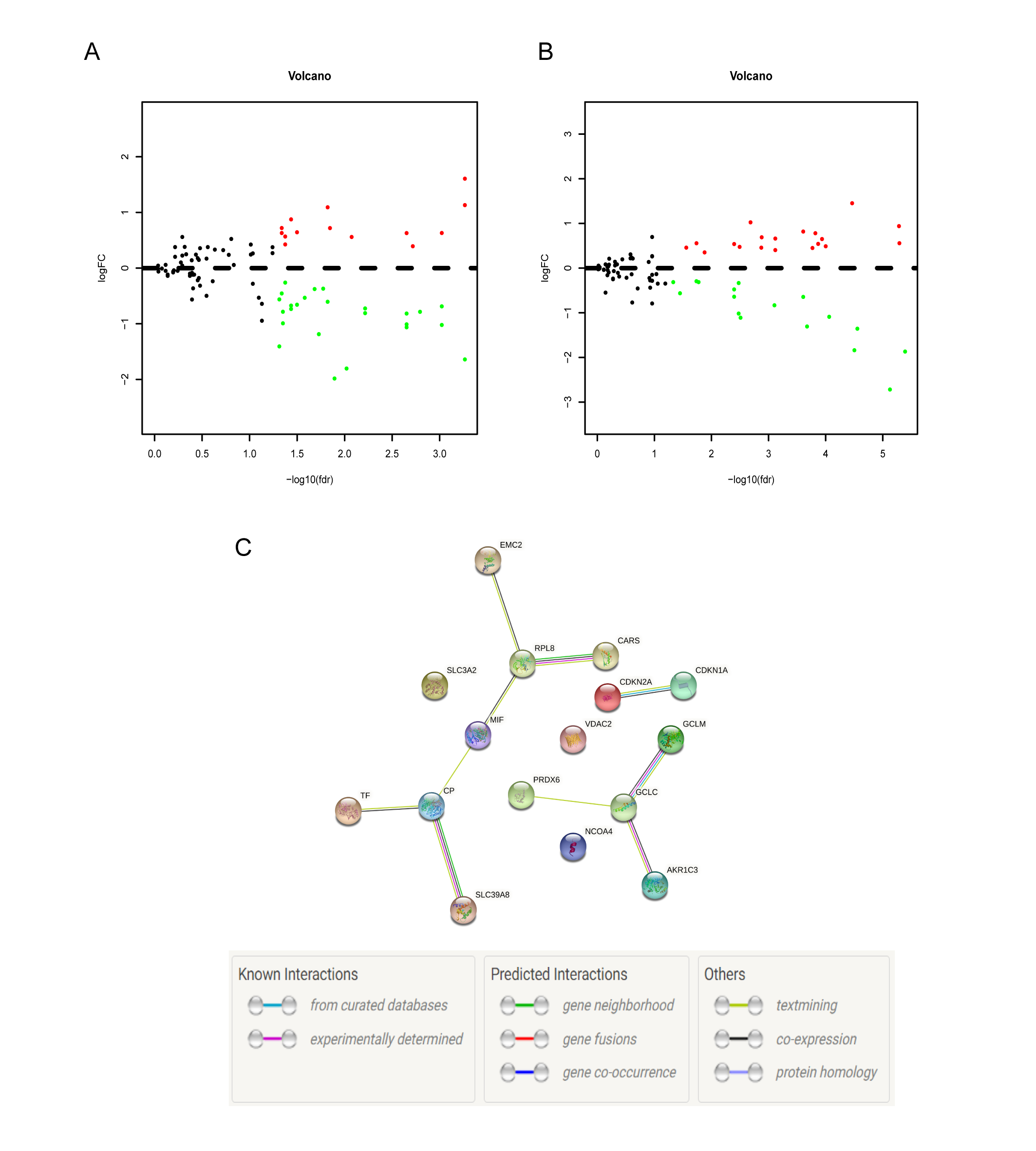
**Supplementary Figure 2. (A)** The volcano plot of differentially expressed FRGs in GSE9782. **(B)** The volcano plot of differentially expressed FRGs in GSE24080. **(C)** PPI network of differentially expressed FRGs.


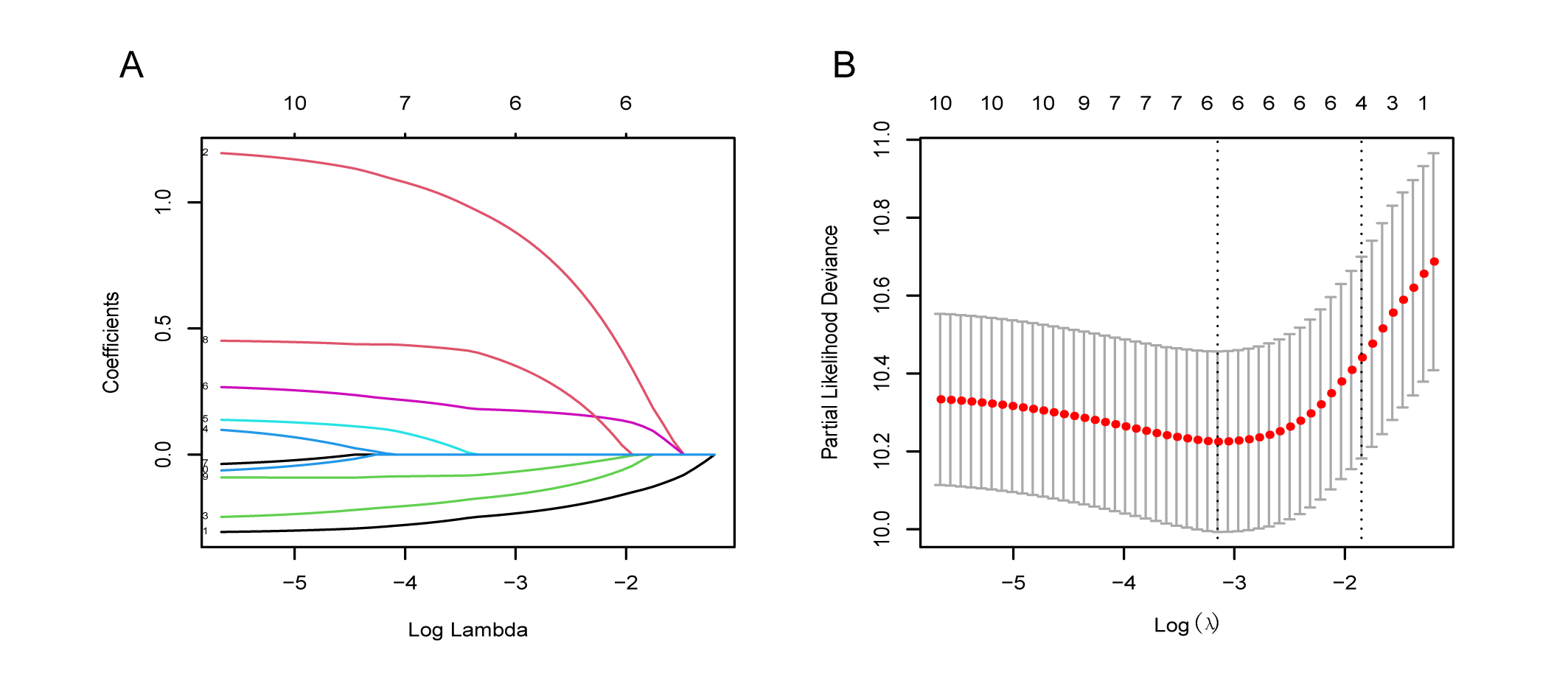


**Supplementary Figure 3. (A)** The LASSO Cox regression analysis identified six FRGs in the training cohort (GSE9782). Each curve corresponds to one gene. **(B)** Partial likelihood deviance of different numbers of variables. One-thousand-fold cross-validation was applied for tuning penalty parameter selection.


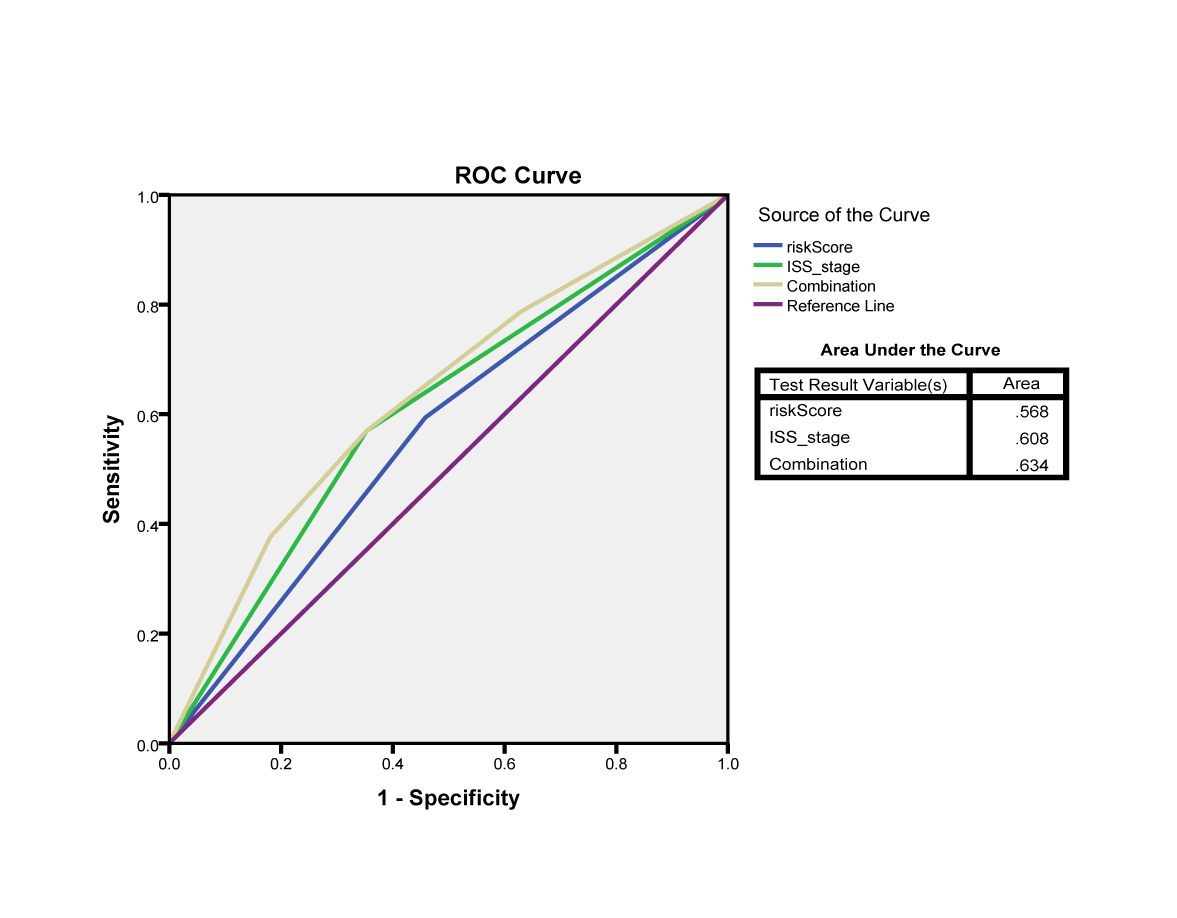


**Supplementary Figure 4.** The ROC curves of the predictive efficacy combined risk score with ISS stage.


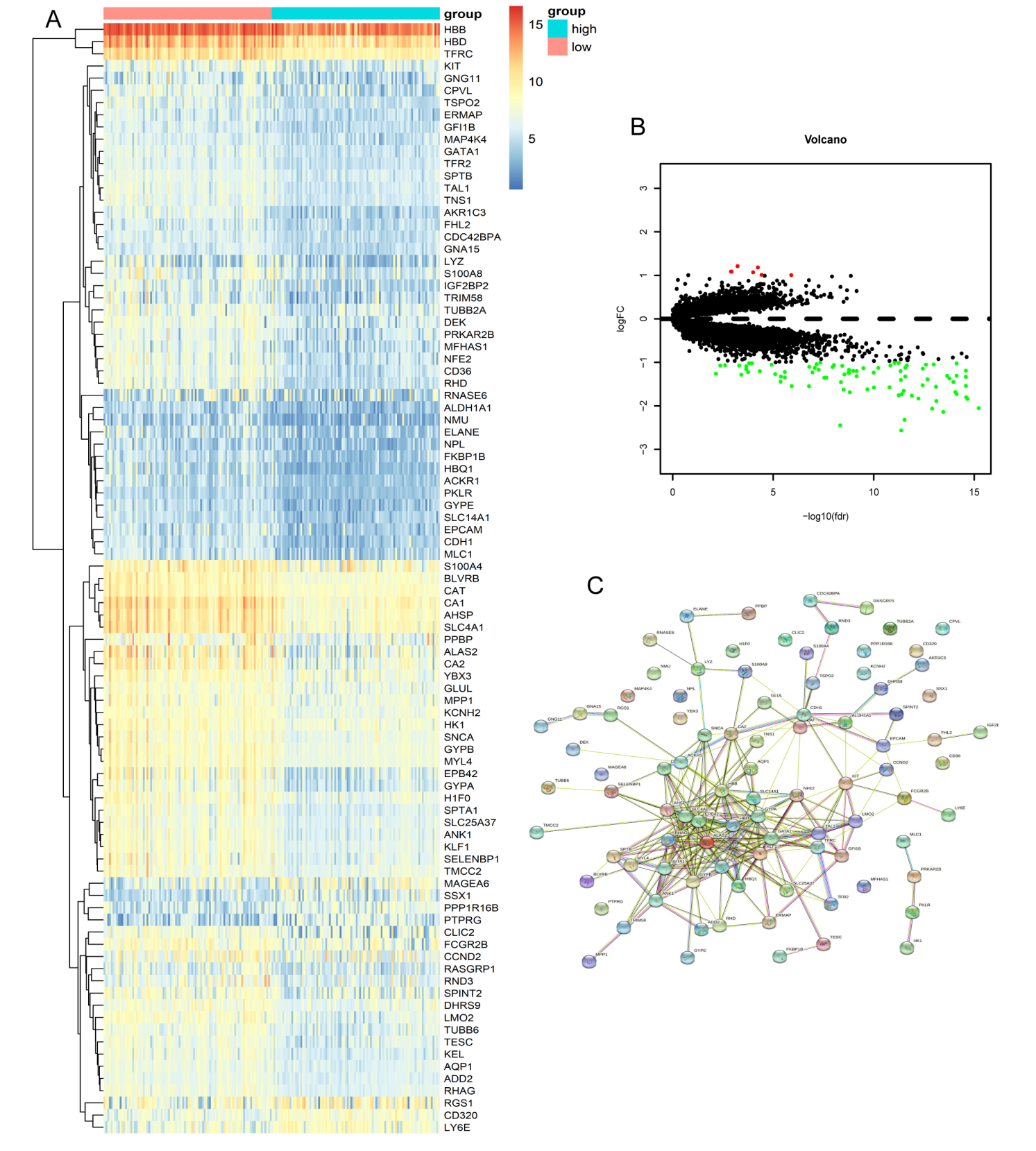


**Supplementary Figure 5. (A)** The heatmap of DEGs between high- and low-risk groups. **(B)** The volcano plot of DEGs between high- and low-risk groups. **(C)** PPI network of DEGs.


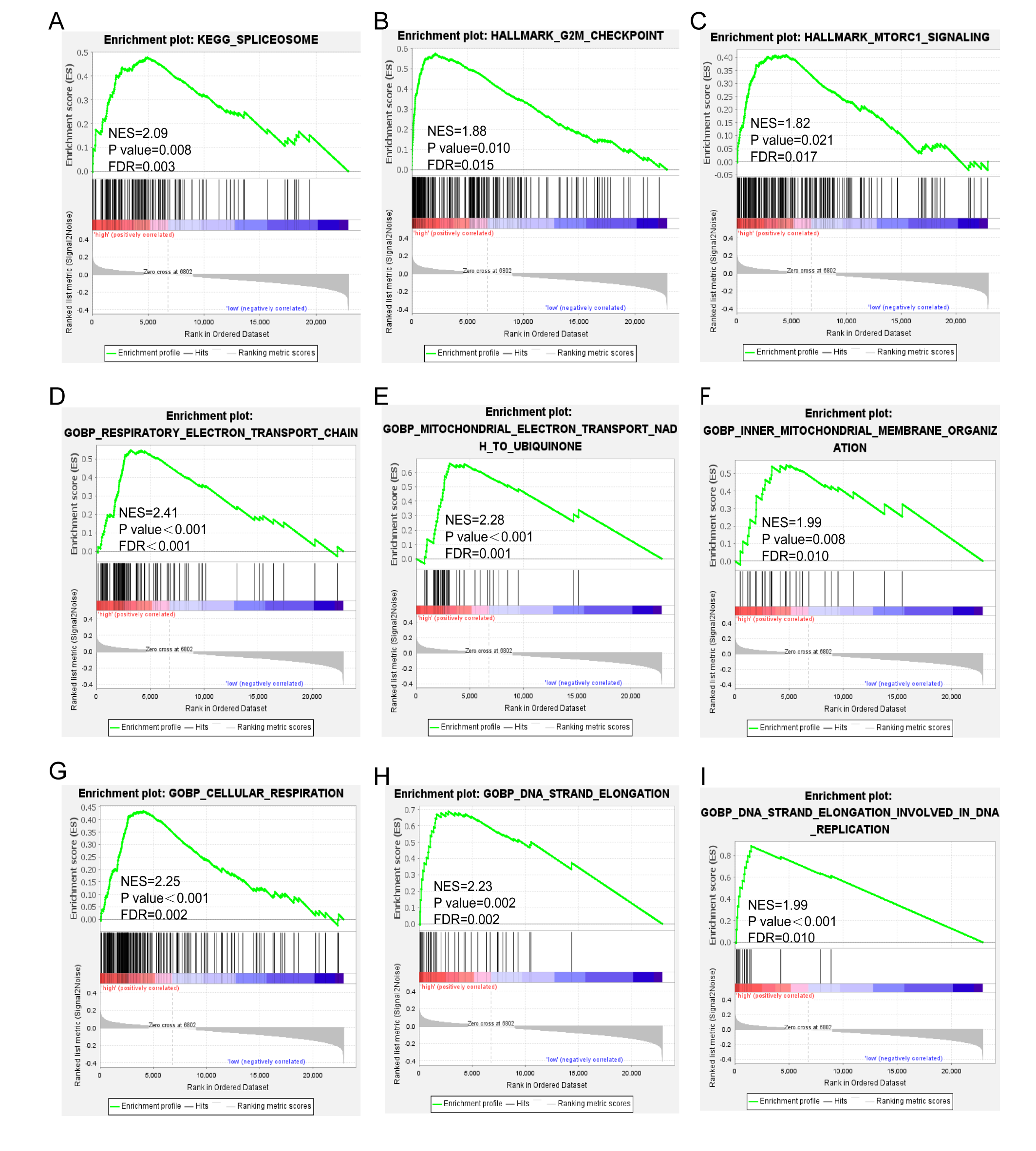


**Supplementary Figure 6. (A-I)** GSEA results of KEGG pathways **(A)**, HALLMARK **(B-C)** and GOBP **(D-I)** in GSE24080.





**Supplementary Figure 7.** Days to progression of MM patients in high and low risk groups in GSE9782. (**p=0.031)


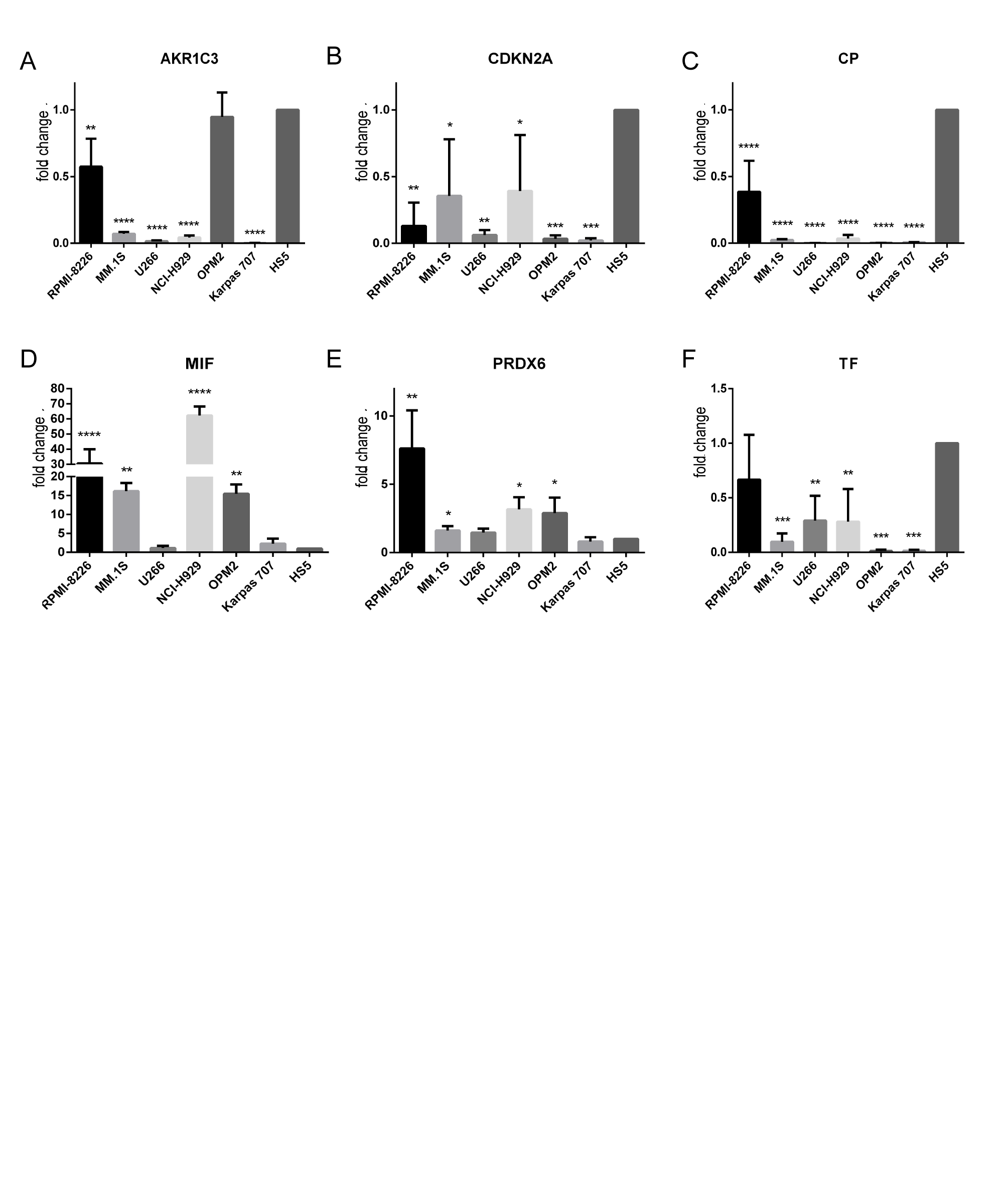


**Supplementary Figure 8. Validations for FRGs expressions in MM cell lines. (A-F)** AKR1C3, CDKN2A, CP, MIF, PRDX6 and TF mRNA expression in MM cell lines (RPMI-8226, MM.1S, U266, NCI-H929, OPM2, Karpas 707) vs HS5 cell. *p＜0.05, **p＜0.01, ***p＜0.001, ****p＜0.0001.


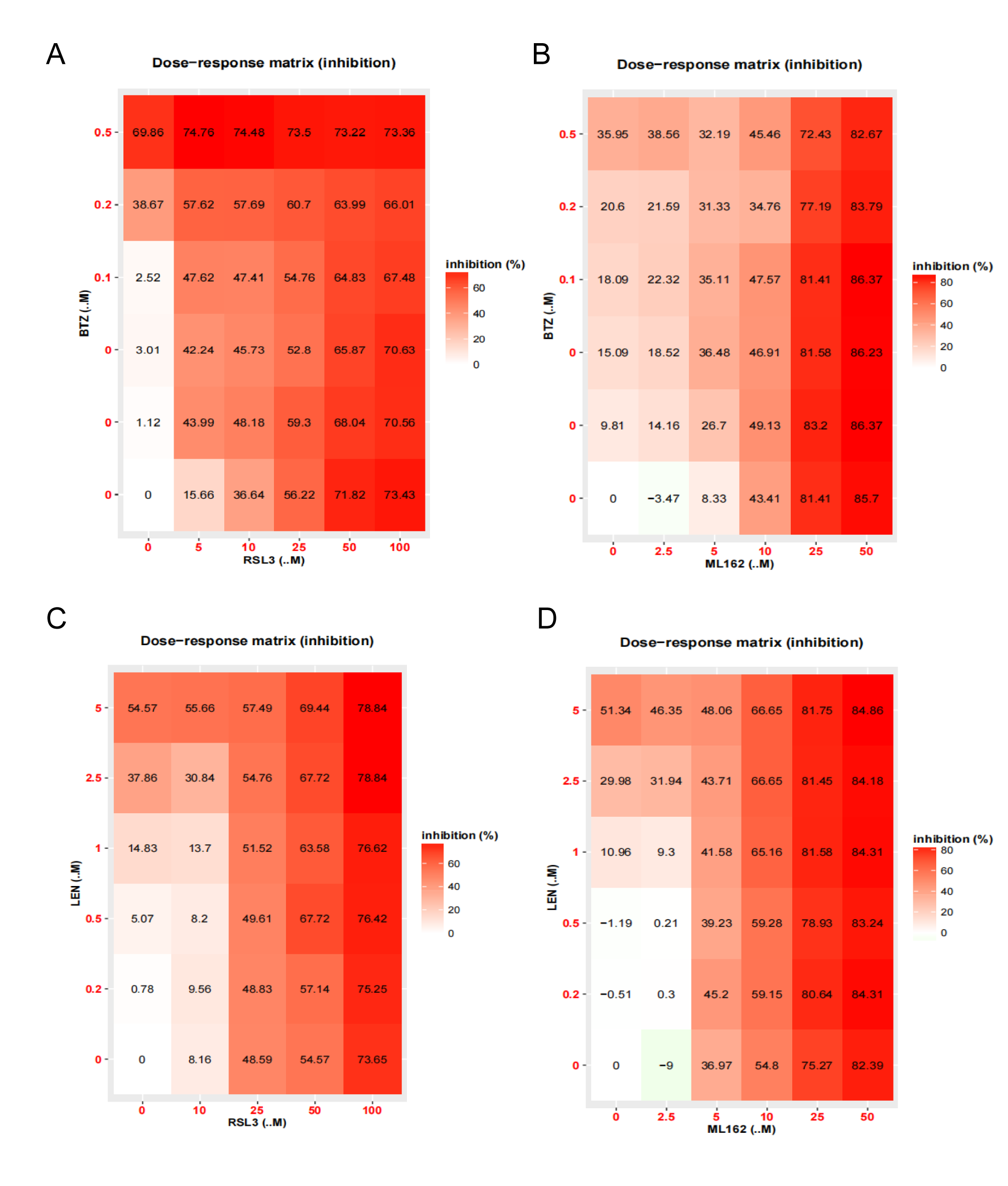

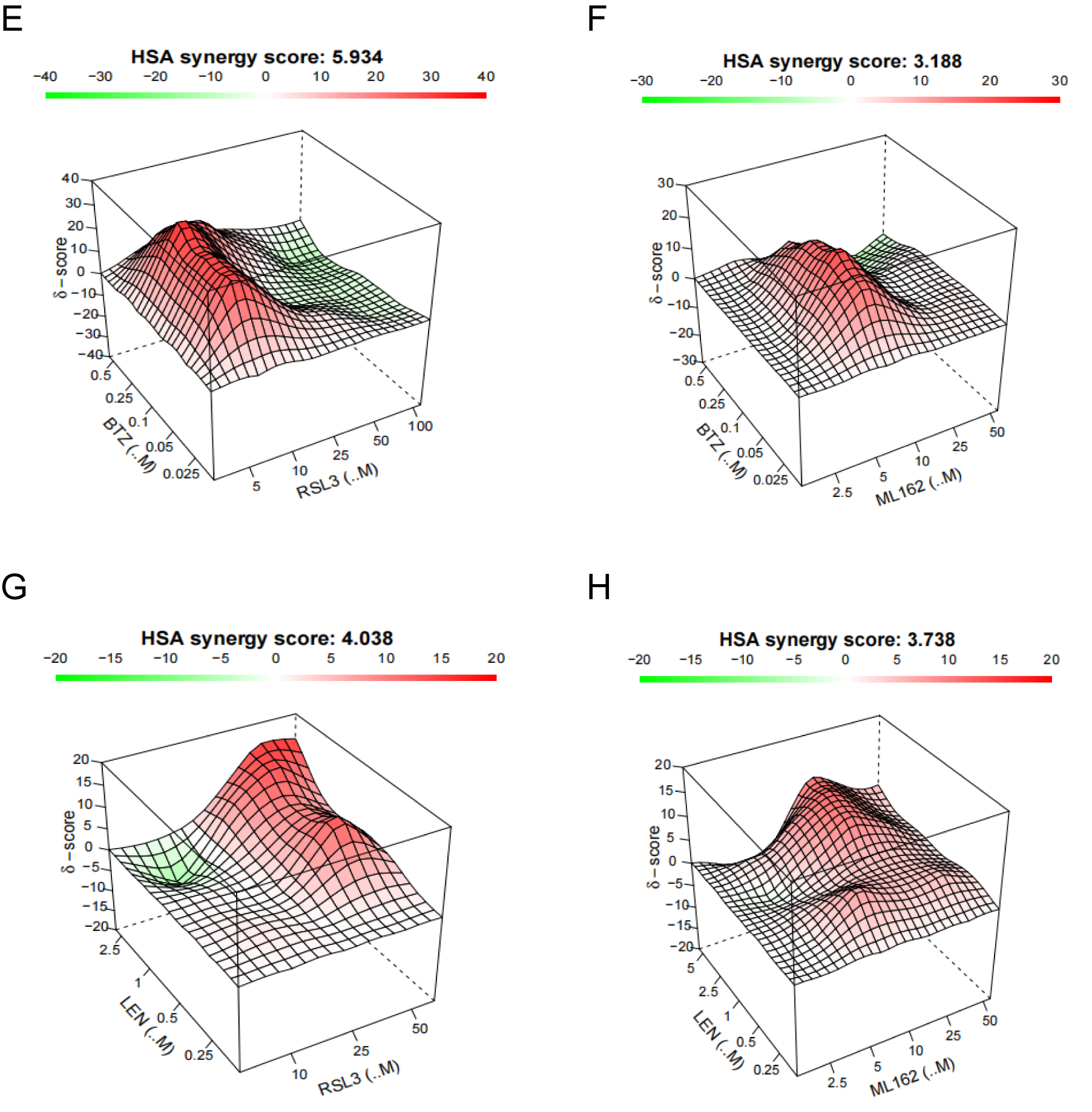


**Supplementary Figure9. (A-D) Dose-response matrix of BTZ+RSL3 (A), BTZ+ML162 (B), LEN+RSL3 (C), LEN+ML162 (D) in RPMI-8226 cell line. (E-H) The 3D plot of BTZ+RSL3 (E), BTZ+ML162 (F), LEN+RSL3 (G), LEN+ML162 (H) in RPMI-8226 cell line.**


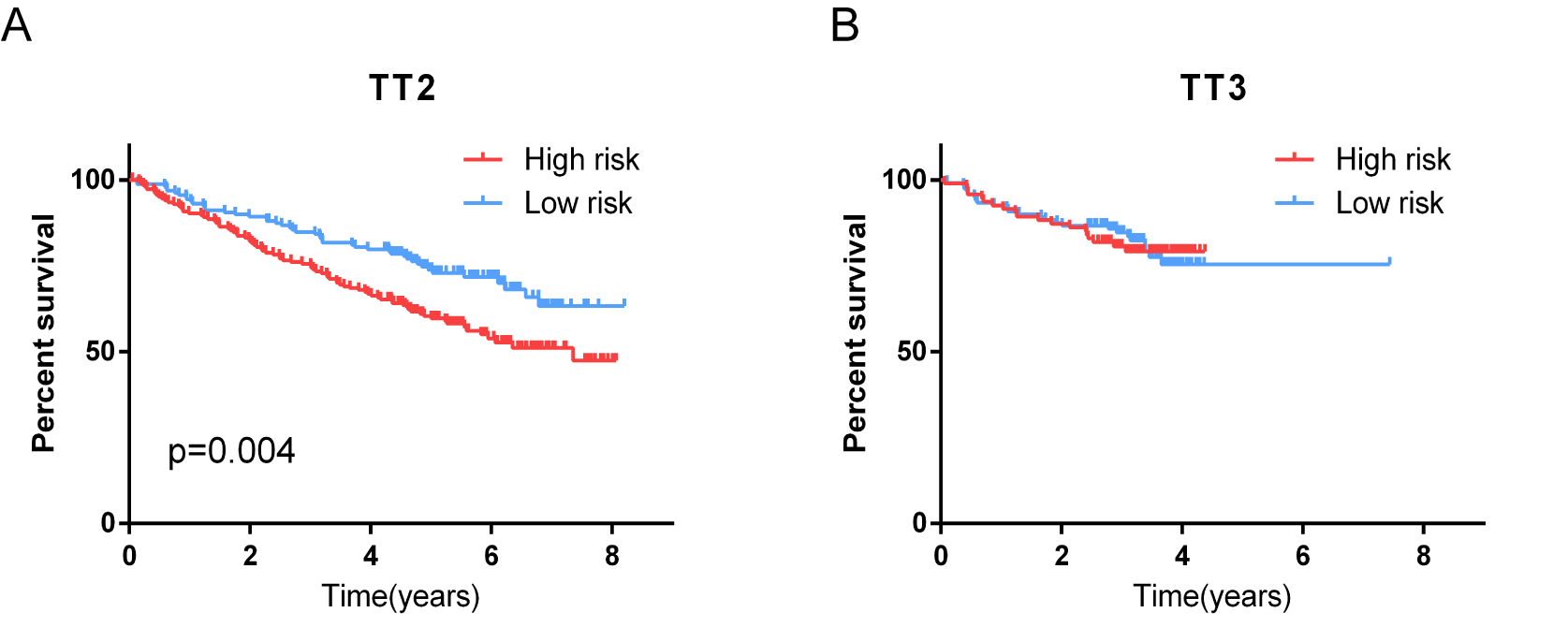


**Supplementary Figure10. Validation of FRGs risk signature in different treatment subgroups in GSE24080. (A-B)** Kaplan-Meier survival curves for the OS of MM patients in the TT2 (thalidomide, n=351) and TT3 (bortezomib, n=214) subgroups in GSE24080.
